# Macrotrabecular-massive subtype in hepatocellular carcinoma based on contrast-enhanced CT: deep learning outperforms machine learning

**DOI:** 10.1186/s13244-025-02063-w

**Published:** 2025-08-28

**Authors:** Lulu Jia, Zeyan Li, Gang Huang, Hanchen Jiang, Hao Xu, Jianxin Zhao, Jinkui Li, Junqiang Lei

**Affiliations:** 1 The First Clinical Medical College of Lanzhou University, Lanzhou, China; 2 Jinan University & University of Birmingham Joint Institution, Jinan University, Jinan, China; 3 Department of Radiology, Gansu Provincial Hospital, Lanzhou, China; 4 Department of Statistics, University of California, Los Angeles, Los Angeles, CA USA; 5 Department of Medicine, Harvard Medical School, Brigham and Women’s Hospital, Boston, MA USA; 6 Department of Radiology, Gansu provincial cancer hospital, Lanzhou, China; 7 Department of Radiology, The First Hospital of Lanzhou University, Lanzhou, China

**Keywords:** Hepatocellular carcinoma, Deep learning, Macrotrabecular-massive subtype, CT

## Abstract

**Objective:**

To develop a CT-based deep learning model for predicting the macrotrabecular-massive (MTM) subtype of hepatocellular carcinoma (HCC) and to compare its diagnostic performance with machine learning models.

**Materials and methods:**

We retrospectively collected contrast-enhanced CT data from patients diagnosed with HCC via histopathological examination between January 2019 and August 2023. These patients were recruited from two medical centers. All analyses were performed using two-dimensional regions of interest. We developed a novel deep learning network based on ResNet-50, named ResNet-ViT Contrastive Learning (RVCL). The RVCL model was compared against baseline deep learning models and machine learning models. Additionally, we developed a multimodal prediction model by integrating deep learning models with clinical parameters. Model performance was evaluated using the area under the receiver operating characteristic curve (AUC).

**Results:**

A total of 368 patients (mean age, 56 ± 10; 285 [77%] male) from two institutions were retrospectively enrolled. Our RVCL model demonstrated superior diagnostic performance in predicting MTM (AUC = 0.93) on the external test set compared to the five baseline deep learning models (AUCs range 0.46–0.72, all *p* < 0.05) and the three machine learning models (AUCs range 0.49-0.60, all *p* < 0.05). However, integrating the clinical biomarker Alpha-Fetoprotein (AFP) into the RVCL model did not significant improvement in diagnostic performance (internal test data set: AUC 0.99 vs 0.95 [*p* = 0.08]; external test data set: AUC 0.98 vs 0.93 [*p* = 0.05]).

**Conclusion:**

The deep learning model based on contrast-enhanced CT can accurately predict the MTM subtype in HCC patients, offering a smart tool for clinical decision-making.

**Critical relevance statement:**

The RVCL model introduces a transformative approach to the non-invasive diagnosis MTM subtype of HCC by harmonizing convolutional neural networks and vision transformers within a unified architecture.

**Key Points:**

- The RVCL model can accurately predict the MTM subtype.
- Deep learning outperforms machine learning for predicting MTM subtype.
- RVCL boosts accuracy and guides personalized therapy.

**Graphical Abstract:**

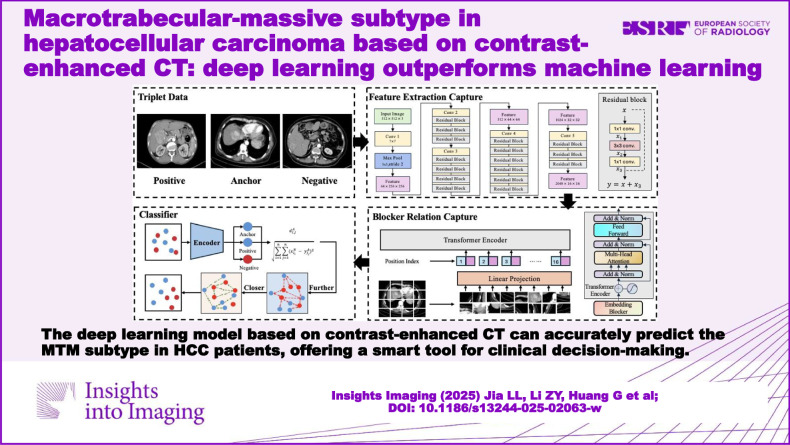

## Introduction

The macrotrabecular-massive (MTM), a subtype of hepatocellular carcinoma (HCC), was formally recognized by the World Health Organization in 2019 [1–3]. It is characterized by trabecular structures exceeding six cells in thickness [4–6]. Compared to non-MTM HCC, the MTM subtype typically presents with larger tumor volumes, elevated serum alpha-fetoprotein (AFP) levels, and a significantly worse prognosis [7]. MTM-HCC may be somewhat susceptible to angiogenesis inhibitors, such as anti-Ang-2 and anti-VEGFA antibodies, due to its distinct molecular kinds and carcinogenic pathways [8]. Additionally, researchers have discovered that CKLF-like MARVEL transmembrane domain-containing 6 (CMTM6) stimulates the production of Programmed Death-1 (PD-L1) in tumor cells as a defensive mechanism against T lymphocytes. Therefore, immune status evaluation in conjunction with anti-CMTM6 and anti-PD-L1 therapy may be more successful in treating MTM-HCC [9, 10]. Consequently, the identification of MTM-HCC prior to treatment initiation holds substantial clinical significance for tailoring personalized therapeutic approaches and predicting patient outcomes. However, current assessment of MTM relies on pathological specimens, which can introduce potential sampling errors and necessitate postoperative evaluation.

Traditional imaging features have been explored for predicting MTM. Specifically, arterial phase (AP) hypoenhancement exceeding 20% or 50%, extensive necrosis, and severe ischemia have been identified as independent predictors of MTM. However, their sensitivity (7.4–57%) and specificity (31–66%) remain suboptimal [11–14]. Beyond imaging diagnostics, a growing number of research studies suggest that radiomics models can effectively predict MTM [7, 15–25]. Nevertheless, the performance of these models in test sets remains unsatisfactory, with specificity (SPE, < 62%) and positive predictive value (PPV, < 46%) remaining particularly low [15]. Furthermore, significant variability exists across studies, with some identifying random forest (RF) as the best-performing model, others favoring support vector machines (SVM), and yet others demonstrating the superiority of logistic regression (LR) models over the former two [15, 18, 23]. This inconsistency has raised widespread concerns regarding the standardization, reproducibility, stability, and clinical translatability of radiomics models.

Deep learning (DL) approaches have advanced medical image diagnosis by addressing the limitations of traditional machine learning (ML) algorithms [26–28]. Transformer-based architectures have demonstrated potential in handling medical image diagnosis due to their ability to effectively capture global feature dependencies [29–31]. A recent study [25] developed a multitask DL radiomics model for predicting both MTM subtype and prognosis in HCC patients after hepatic arterial infusion chemotherapy (HAIC). However, this approach was limited by its requirement for post-HAIC patient data. We therefore propose that our novel attention-based DL model can provide more accurate preoperative prediction of MTM-HCC.

Hence, this study aims to develop a novel DL model in predicting the MTM subtype in HCC patients, using postoperative histopathological findings as the gold standard. Additionally, a comprehensive comparative analysis will be conducted to evaluate the proposed model’s diagnostic performance against existing DL and traditional ML models, ensuring a rigorous assessment of its accuracy, robustness, and clinical applicability.

## Materials and methods

### Study population

The study received approval from the Institutional Review Board of all participating centers, and due to its retrospective nature, the requirement for informed consent was waived (LDYYLL-2024-398, 2022-432). The training data set and internal test data set were derived from patients at the First Clinical Hospital of Lanzhou University (Center 1), while the external test data set comprised patients from Gansu Provincial People’s Hospital (Center 2).

**Fig. 1:**
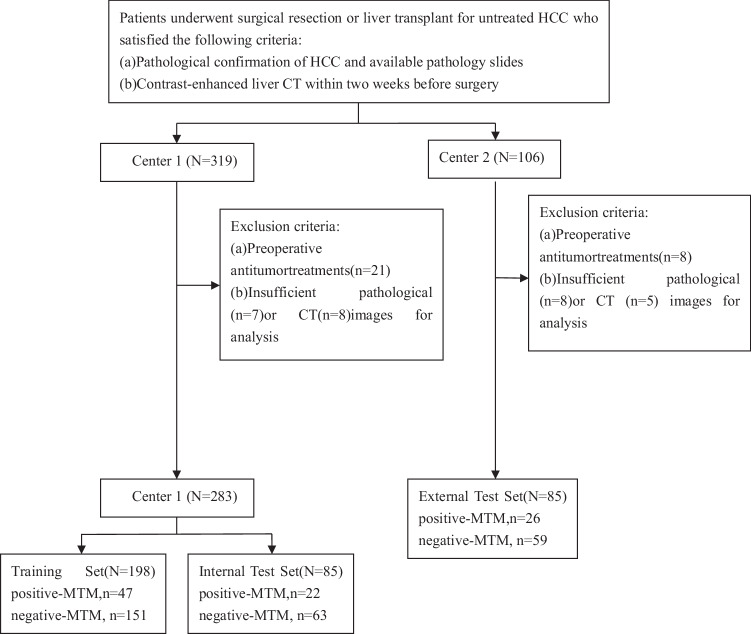
Flowchart of patient inclusion and exclusion in this retrospective multicenter study. From two centers, 425 treatment-naive HCC patients (Center 1: *N* = 319; Center 2: *N* = 106) with available pathology and pretreatment CT were initially screened. After excluding 21 patients (Center 1) and 8 patients (Center 2) who received preoperative antitumor therapy, plus 23 (Center 1) and 13 (Center 2) with inadequate imaging/pathology data, 368 patients were included (Center 1: *N* = 283; Center 2: *N* = 85). The cohort was divided into training (*N* = 198; positive-MTM = 47, negative-MTM = 151), internal test (*N* = 85; positive-MTM = 22, negative-MTM = 63), and external test (*N* = 85; positive-MTM = 26, negative-MTM = 59) sets for model development and validation. HCC, hepatocellular carcinoma; MTM, macrotrabecular-massive

Consecutive patients who underwent surgical resection or liver transplantation for HCC between January 2019 and August 2023 were included. Patients were eligible if they met the following criteria: (1) age greater than 18 years; (2) contrast-enhanced CT of the liver performed within two weeks prior to surgery; (3) histopathological confirmation of HCC; and (4) availability of clinical laboratory data and pathological slides. Exclusion criteria included: (1) a history of local regional therapy or chemotherapy prior to surgery; and (2) poor image quality or artifacts (Fig. 1).

For model training, patients from Center 1 were randomly sampled for the training dataset and internal test dataset in a 7:3 ratio. The dataset from Center 2 was exclusively used as the external test dataset for independent validation. To ensure consistency and comparability, all models were trained and evaluated using the same datasets.

### Clinical and laboratory data

Demographic characteristics and clinical data were collected, including age, gender, etiology of liver disease (hepatitis C, hepatitis B, or others), tumor size, AFP level, platelet count, neutrophil count, neutrophil-to-lymphocyte ratio (NLR), prothrombin time (PT), international normalized ratio (INR), aspartate aminotransferase (AST) level, alanine aminotransferase (ALT) level, albumin level, total bilirubin level, and carcinoembryonic antigen (CEA) level.

## Histologic diagnosis and immunohistochemistry

### Pathological evaluation

All histological specimens were reviewed by abdominal pathologists who were blinded to other clinical and imaging data. Tumors with a predominant (> 50%) macrotrabecular architecture, characterized by trabecular cords exceeding six cells in thickness, were classified as MTM [3]. Additional histopathological assessments were conducted, including the determination of microvascular invasion (MVI) status.

### CT imaging

CT images were acquired in the transverse plane, covering both arterial and portal venous phases. Detailed imaging protocols are outlined in Tables S1 and S2.

### Radiomics analysis

A board-certified abdominal radiologist (Reader 1, L.J., with 6 years of experience in liver imaging) manually segmented two-dimensional regions of interest (ROIs) on axial arterial phase (AP) and portal venous phase (PVP) images for HCC lesions across all three datasets, focusing on the axial slice with the largest tumor cross-section using ITK-SNAP software (http://www.itksnap.org). All segmentations were then verified by a senior radiologist (J.K.). To assess intra- and inter-observer reproducibility, Reader 1 and another radiologist (J.Q.) independently re-segmented a randomly selected subset of 60 cases one month later, with intraclass correlation coefficients (ICCs) calculated to evaluate consistency.

Subsequently, the ROIs from both arterial and portal venous phases were imported into the “FeAture Explorer (FAE) v.0.5.7” software (https://github.com/salan668/FAE.git) to extract a comprehensive set of quantitative features. In addition to raw features, advanced transformations—including Wavelet Transform, Square, Square Root, Logarithm, Laplacian of Gaussian, Gradient, and Exponential filters—were applied to each ROI to derive radiomics features. Features with an ICC below 0.8 were considered non-reproducible and excluded from further analysis.

The radiomics model was developed in the training cohort through the following steps: (a) each feature was standardized using *Z*-score normalization; (b) Pearson correlation coefficients (PCC) were estimated for each feature pair, and collinear features with a threshold exceeding 0.90 were eliminated; (c) analysis of variance (ANOVA) was employed to select candidate features; and (d) various classifiers, including SVM, RF, and LR, were utilized to construct diagnostic models. All steps were performed using 5-fold cross-validation on the training dataset to identify the optimal combination yielding the highest AUC.

### DL model development

**Fig. 2:**
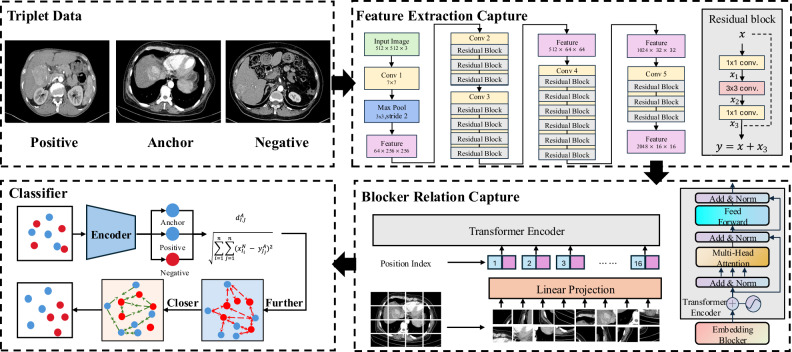
The structure of our proposed model for classification. Architecture overview of the proposed DL framework, illustrating the triplet data formulation, feature extraction pipeline, and classification module. The triplet component demonstrates positive and negative sample relationships using distance metrics (dij) and feature difference calculations (∑(xi²-yi²)²). The feature extraction module processes 512 × 512 × 5 input images through a series of convolutional layers (Conv1, Conv2), max pooling, and residual blocks to generate 64 × 256 × 256 feature maps. The classifier incorporates a transformer encoder with position indexing and linear projection, followed by multi-scale feature processing through sequential 3 × 1 and 3 × 3 convolutional layers, ultimately transforming 512 × 64 × 64 features into 1024 × 32 × 32 representations for final prediction

This section delineates the methodology of our proposed learning framework (illustrated in Fig. 2) for tumor classification, which integrates convolutional neural networks (CNNs) with vision transformers (ViTs) to achieve effective feature extraction, fusion, and discrimination. The axial CT slice demonstrating the largest tumor diameter served as the input to the DL model. A comprehensive description of the process is provided in Appendix S1.

For comparative analysis, the same sample size was used to train and validate five distinct models: AlexNet [32], VGG [33], ResNet [34], ViT [35], and EfficientNet [36].

### Statistical analysis

Baseline characteristics were summarized as follows: continuous variables were reported as mean ± standard deviation if they met the assumptions of normality and homogeneity of variance; otherwise, they were reported as median and interquartile range (IQR). Categorical variables were presented as frequencies and percentages. For comparisons of continuous variables, independent samples *t*-tests were used when normality and homogeneity of variance assumptions were met; otherwise, the Mann–Whitney *U*-test was applied. Categorical variables were analyzed using the chi-square (χ^2^) test. To assess differences in continuous variables across the training, internal test, and external test datasets, one-way analysis of variance (ANOVA) or the Kruskal–Wallis test was applied, depending on the data distribution. Post-hoc pairwise comparisons were conducted with Bonferroni correction to control for multiple testing. The predictive performance of the models was evaluated using receiver operating characteristic (ROC) curve analysis, with the AUC calculated and compared using the DeLong method.

## Results

### Patient characteristics

Among the 427 patients initially screened, 59 were excluded based on predefined criteria (Fig. 1), resulting in a final cohort of 368 patients. This cohort included 198 patients (54%) in the training dataset (mean age, 56 ± 10 years [SD]; 156 males [79%], 42 females [21%]), 85 patients (23%) in the internal test dataset (mean age, 57 ± 9 years [SD]; 63 males [74%], 22 females [26%]), and 85 patients in the external test dataset (mean age, 57 ± 11 years [SD]; 66 males [78%], 19 females [22%]). Hepatitis B virus infection, as the predominant risk factor for liver disease, was present in 287 of the 368 patients (78%). When laboratory variables were analyzed as continuous measures, all characteristics exhibited similar distributions across the training, internal, and external test datasets, except for AFP levels (*p* = 0.002) and international normalized ratio (INR) (*p* = 0.002) (Table 1). In the training set, 47 of the 198 patients (24%) were diagnosed with MTM-HCC. Compared with non-MTM HCC patients, these MTM patients exhibited significantly higher serum AFP levels (*p* = 0.002) (Table 2).

**Table 1:** Clinical and pathologic characteristics of patients with HCC in the training, internal test, and external test sets

Characteristics	Training data set (*n* = 198)	Internal test data set (*n* = 85)	External test data set (*n* = 85)	*p* value
Age (y)*	56 ± 10	57 ± 9	56 ± 11	0.86
Sex				0.69
Male	156 (79)	63 (74)	66 (78)	
Female	42 (21)	22 (26)	19 (22)	
Etiology				0.06
HBV infection	158 (80)	65 (77)	57 (67)	
HCV infection	5 (3)	5 (6)	2 (2)	
Other	35 (18)	15 (18)	26 (31)	
Serum AFP level^‡^	10.0 (3.2–196.3)	8.7 (3.6–238.5)	174.0 (3.9–1000.0)	0.002^ǁ^
Platelet count (×10^9^/L)^‡^	135.0 (94.0–186.0)	127.0 (90–181.8)	139.0 (98.5–196.5)	0.57
Neutrophil count (^9/L) ^‡^	3.8 (2.6–6.3)	3.6 (2.6–6.2)	3.9 (2.7–5.9)	0.95
Lymphocyte count (^9/L)	1.0 (0.7–1.3)	1.1 (0.8–1.5)	1.1 (0.7–1.6)	0.14
The neutrophil-to-lymphocyte ratio^‡^	3.7 (2.2–7.2)	3.1 (2.1–6.4)	3.3 (2.4–4.7)	0.62
Prothrombin time (s)^‡^	13.2 (12.1–15.0)	13.0 (12.2–14.5)	13.6 (12.6–14.9)	0.11
INR^‡^	1.2 (1.1–1.3)	1.2 (1.1–1.4)	1.1 (1.0–1.2)	0.002^ǁ^
AST level (IU/L)^‡^	52.0 (32.0–103.8)	49.5 (33.0–88.0)	45.0 (28.8–77.5)	0.29
ALT level (IU/L)^‡^	61.0 (33.0–127.0)	63.5 (40.3–123.8)	51.0 (28.0–85.3)	0.19
ALB level (g/L)^‡^	35.5 (32.7–40.1)	35.0 (32.2–39.1)	36.7 (32.7–40.6)	0.47
Total bilirubin level (umol/L)^‡^	23.2 (16.2–34.7)	24.3 (17.4–36.3)	21.1 (14.5–28.0)	0.15
Size (cm)*	5.0 ± 3.2	4.6 ± 2.9	4.7 ± 2.8	0.24
CEA	1.9 (1.2–3.3)	2.2 (1.6–3.2)	2.4 (1.6–3.5)	0.11
MTM	47 (24)	22 (26)	26 (31)	0.48

Unless indicated otherwise, data are numbers of patients, with percentages in parentheses

*AFP* α-fetoprotein, *ALB* albumin, *ALT* alanine aminotransferase, *AST* aspartate aminotransferase, *CEA* Carcino-embryonic antigen, *HBV* hepatitis B virus, *HCV* hepatitis C virus, *INR* International normalized ratio, *MVI* microvascular invasion

* Data are means ± SDs

^‡^ Data are medians, with IQRs in parentheses

^ǁ^ Statistical significance was found between the training and external test data sets and the internal and external test data sets

**Table 2:** Patient characteristics in the training data set according to the MTM subtype

Characteristics	MTM (*n* = 47)	Non-MTM (*n* = 151)	*p* value
Age (y)^*^	55 ± 9	57 ± 10	0.224
Sex			0.498
Male	30 (64)	88 (58)	
Female	17 (36)	63 (42)	
Etiology			0.940
HBV infection	37 (79)	121 (80)	
HCV infection	1 (2)	4 (3)	
Others	9 (19)	26 (17)	
Serum AFP level^‡^	72.1 (6.8–444.5)	7.0 (2.7–159.0)	0.002
Platelet count (×10^9^/L)^‡^	131.0 (96.0–179.0)	137.0 (92.8–187.3)	0.777
Neutrophil count (^9/L) ^‡^	3.7 (2.5–7.4)	3.9 (2.6–6.1)	0.851
The neutrophil-to-lymphocyte ratio^‡^	4.0 (2.3–8.0)	3.5 (2.2–7.2)	0.476
Prothrombin time (s)^‡^	13.6 (12.2–15.1)	13.1 (12.0–14.9)	0.362
INR^‡^	1.2 (1.1–1.3)	1.2 (1.1–1.3)	0.199
AST level (IU/L)^‡^	52.0 (31.0–101.0)	52 (32.0–115.5)	0.803
ALT level (IU/L)^‡^	60.0 (28.0–113.0)	63.0 (34.5–136.5)	0.411
ALB level (g/L)^‡^	34.8 (32.3–39.6)	35.6 (32.8–40.1)	0.402
Total bilirubin level (umol/L)^‡^	24.9 (15.1–30.1)	22.5 (16.2–35.9)	0.666
Size (cm)^*^	4.5 (2.5–7.6)	3.9 (2.6–6.7)	0.605
MVI	32 (68)	86 (57)	0.220
CEA^‡^	1.9 (1.0–3.4)	2.0 (1.2–3.3)	0.726

Unless indicated otherwise, data are numbers of patients, with percentages in parentheses

*AFP* α-fetoprotein, *ALB* albumin, *ALT* alanine aminotransferase, *AST* aspartate aminotransferase, *CEA* Carcino-embryonic antigen, *HBV* hepatitis B virus, *HCV* hepatitis C virus, *INR* International normalized ratio, *MVI* microvascular invasion

^*^ Data are means ± SDs

^‡^ Data are medians, with IQRs in parentheses

### Analysis of clinical factors

Continuous laboratory variables were dichotomized based on the normal ranges or clinical relevance. When laboratory variables were evaluated as categorical measures, an AFP level greater than 100 ng/mL in the training dataset emerged as an independent predictor of MTM HCC (odds ratio [OR] = 1.001 [95% CI: 1.000, 1.002]; *p* = 0.027) (Table S4).

### Diagnostic performance of the ResNet-ViT contrastive learning (RVCL) model

**Fig. 3:**
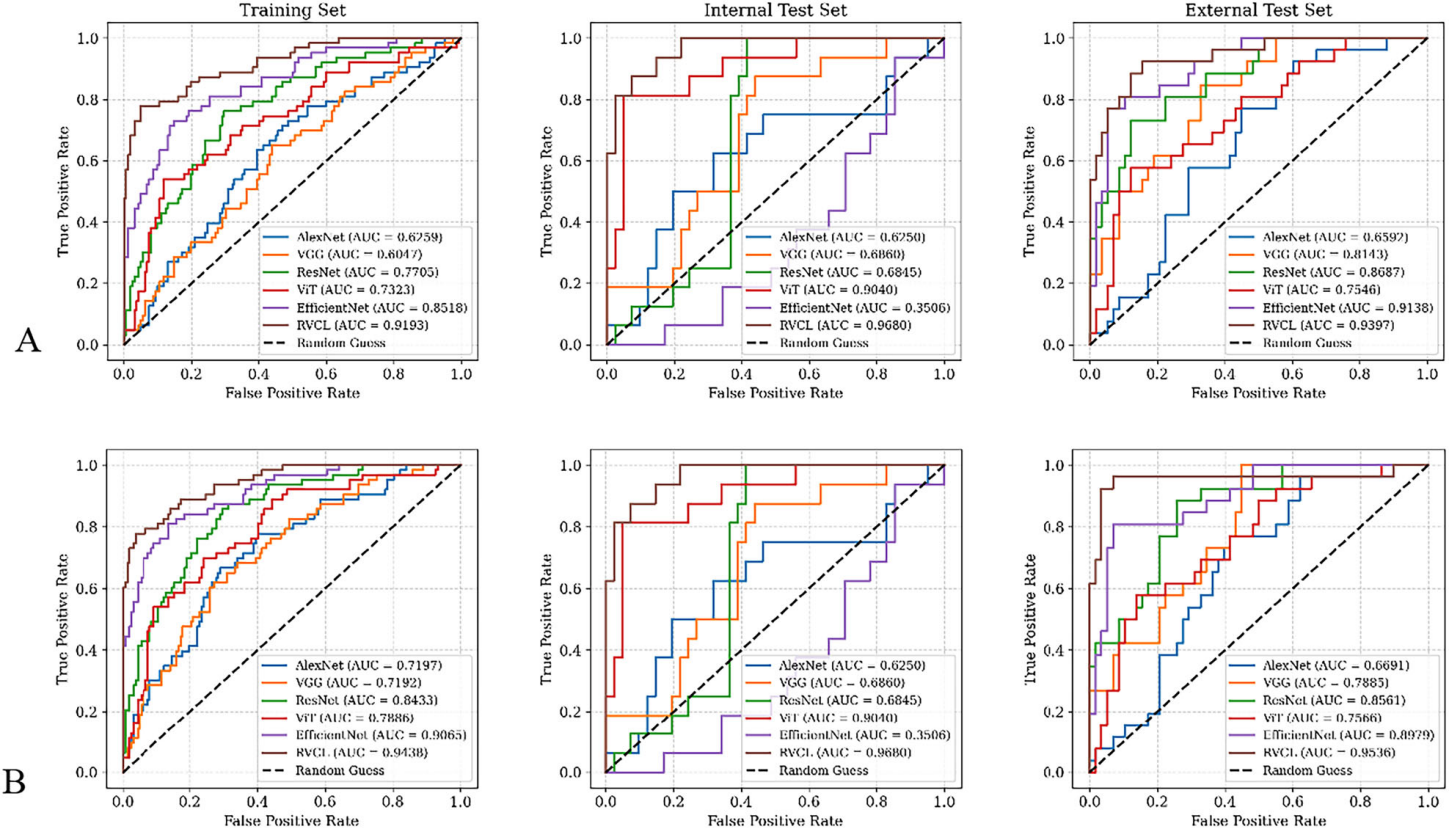
ROC curves for various DL models on the training set, internal test set, and external test set. Each curve represents the true positive rate (TPR) vs the false positive rate (FPR) for different models, with the AUC values indicated. **A** The DL model alone. **B** The model with the clinical factors

We evaluated the performance of our RVCL algorithm using both internal and external test data sets. The results are summarized in Table 3, which presents the detailed metrics (AUC, accuracy, sensitivity, specificity, precision, and F1 score), while the ROC curves are shown in Fig. 3. For the internal test dataset, the model achieved an AUC of 0.95 (95% CI: 0.91, 0.99), with an accuracy of 0.86, sensitivity of 0.75, and specificity of 0.98. On the external test dataset, the model yielded an AUC of 0.93 (95% CI: 0.89, 0.97), with an accuracy of 0.85, sensitivity of 0.75, and specificity of 0.96. When the clinical factor AFP was incorporated into the model, there was no significant difference in AUC (internal test dataset: AUC = 0.99 [95% CI: 0.96, 1.00; *p* = 0.08] and external test dataset: 0.98 [95% CI: 0.94, 1.00; *p* = 0.05]).

**Table 3:** Diagnostic performance of the RVCL and baseline models for predicting MTM-HCC

Model	Training set (*n* = 198)						Internal test set (*n* = 85)							External test set (*n* = 85)					
	Acc	Sen	Spe	Precision	F1 score	AUC	Acc	Sen	Spe		Precision	F1 score	AUC	Acc	Sen	Spe	Precision	F1 score	AUC
AlexNet	0.72	0.52	0.92	0.66	0.46	0.63 [0.55, 0.70]	0.63	0.53	0.81		0.54	0.53	0.51 [0.45, 0.57]	0.60	0.47	0.74	0.46	0.46	0.47 [0.41, 0.53]
VGG	0.73	0.54	0.93	0.69	0.51	0.71 [0.65, 0.77]	0.70	0.55	0.86		0.58	0.54	0.52 [0.46, 0.58]	0.69	0.50	0.94	0.85	0.41	0.46 [0.40, 0.52]
ResNet	0.74	0.52	0.93	0.72	0.52	0.77 [0.72, 0.82]	0.70	0.53	0.84		0.57	0.50	0.61 [0.55, 0.67]	0.68	0.49	0.67	0.34	0.40	0.59 [0.53, 0.65]
ViT	0.75	0.61	0.88	0.69	0.62	0.73 [0.68, 0.78]	0.72	0.50	0.96		0.86	0.42	0.58 [0.52, 0.64]	0.67	0.50	0.79	0.51	0.46	0.52 [0.46, 0.58]
EfficientNet	0.76	0.58	0.96	0.83	0.56	0.79 [0.73, 0.85]	0.74	0.53	0.96		0.87	0.48	0.77 [0.71, 0.83]	0.70	0.52	0.94	0.85	0.45	0.72 [0.66, 0.78]
RVCL	0.88	0.80	0.97	0.91	0.84	0.92 [0.88, 0.96]	0.86	0.75	0.98		0.92	0.79	0.95 [0.91, 0.99]	0.85	0.75	0.96	0.91	0.78	0.93 [0.89, 0.97]
RVCL + C	0.97	0.95	0.99	0.97	0.96	0.98 [0.96, 1.00]	0.96	0.94	0.98		0.98	0.96	0.99 [0.96, 1.00]	0.93	0.88	0.98	0.95	0.91	0.98 [0.94, 1.00]

*AUC* area under the receiver operating characteristic curve, *Acc* accuracy, *C* clinical factor, *DL* deep learning, *HCC* hepatocellular carcinoma, *Sen* sensitivity, *Spe* specificity, *MTM* macrotrabecular-massive, *RVCL* ResNet-ViT contrastive learning, *ViT* vision transformers, *VGG* visual geometry group

### The diagnostic performance of baseline DL models

**Fig. 4:**
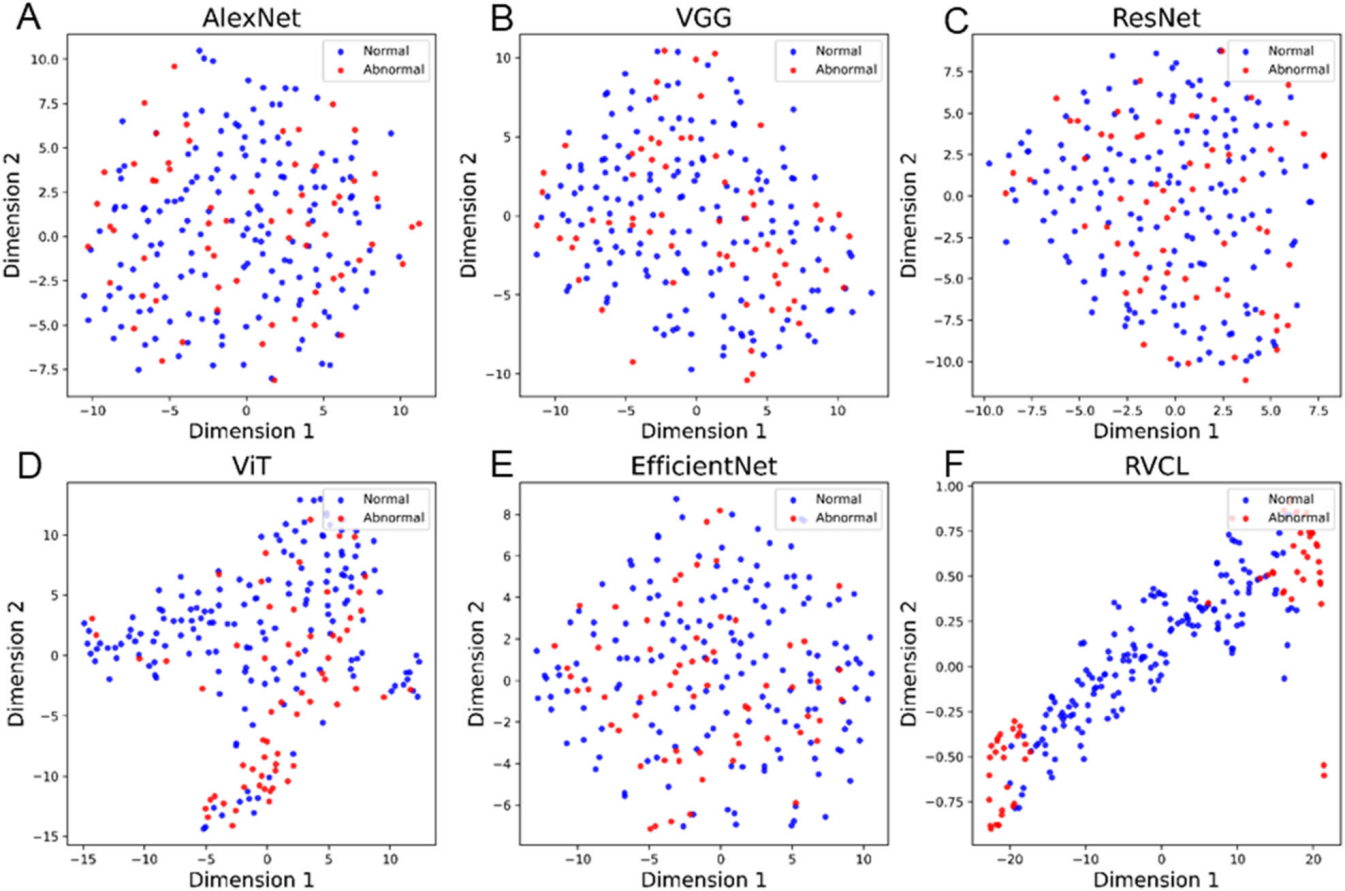
The figure illustrates the two-dimensional embedding spaces generated by various DL models for the identification of the MTM subtype of HCC. The models compared include AlexNet, VGG, ResNet, ViT, EfficientNet, and our newly proposed model, RVCL. Each subplot corresponds to a specific model, with blue dots representing negative MTM cases and red dots representing positive MTM cases. **A** AlexNet: the embedding space shows a relatively scattered distribution of positive and negative MTM cases, indicating limited discriminative power in separating the two classes. **B** VGG: the VGG model demonstrates a slightly improved clustering of positive and negative cases compared to AlexNet, but there is still significant overlap between the two classes. **C** ResNet: ResNet exhibits a more distinct separation between the positive and negative MTM cases, with fewer overlapping regions, suggesting better feature extraction capabilities. **D** ViT: the ViT model shows a clear separation between the two classes, with distinct clusters for positive and negative MTM cases, highlighting its ability to capture complex patterns in the data. **E** EfficientNet: EfficientNet further refines the separation, with well-defined clusters and minimal overlap, indicating its efficiency in learning discriminative features. **F** RVCL (our proposed model): the embedding space generated by our newly proposed RVCL model demonstrates the most distinct separation between positive and negative MTM cases. The clusters are well-defined, with minimal overlap, showcasing the superior performance of our model in identifying the MTM subtype of HCC

The RVCL model demonstrated superior performance across all evaluation metrics compared to existing mainstream models (AlexNet, VGG, ResNet, ViT, and EfficientNet), detailed in Table 3 and Fig. 4 and illustrated in Fig. 3. Among the baselines, AlexNet, VGG, ResNet, and ViT achieved AUC values of 0.63 (95% CI: 0.55, 0.70), 0.71 (95% CI: 0.65, 0.77), 0.77 (95% CI: 0.72, 0.82), and 0.73 (95% CI: 0.68, 0.78), respectively, on the training dataset. However, their AUC values on the internal and external test datasets were close to or below the level of random guessing (0.5). Although EfficientNet had relatively acceptable AUC performance, with values of 0.77 (95% CI: 0.71, 0.83) on the internal test dataset and 0.72 (95% CI: 0.66, 0.78) on the external test dataset, its sensitivity and F1 score were quite low. Specifically, on the internal test dataset, sensitivity and F1 score were 0.53 and 0.48, respectively, while on the external test dataset, they were 0.53 and 0.45, respectively.

### The diagnostic performance of ML models

We finally selected eight radiomic features to input into the models (Appendix S2, Table S3). The three ML models (LR, RF, and SVM) demonstrated significantly inferior performance compared to RVCL across the training, internal test, and external test sets (Table S5). Specifically, LR, RF, and SVM achieved AUC values of 0.53 (95% CI: 0.44, 0.62), 0.53 (95% CI: 0.43, 0.61), and 0.54 (95% CI: 0.45, 0.64), respectively, on the training dataset. Their performance remained largely unchanged on the internal and external test datasets, with AUC values close to or below the level of random guessing (0.5). On the internal test dataset, the AUC values were 0.49 (95% CI: 0.34, 0.64) for LR, 0.50 (95% CI: 0.36, 0.64) for RF, and 0.45 (95% CI: 0.31, 0.58) for SVM. Similarly, on the external test set, the AUC values were 0.60 (95% CI: 0.48, 0.74), 0.49 (95% CI: 0.39, 0.63), and 0.59 (95% CI: 0.46, 0.72), respectively. Notably, the diagnostic performance of the three ML classifiers for identifying the MTM subtype of HCC showed minimal variation.

## Discussion

To achieve a non-invasive and objective diagnosis of the aggressive MTM subtype in HCC, we developed and validated a DL model (RVCL algorithm) based on contrast-enhanced CT, systematically evaluating its diagnostic performance for MTM. The results demonstrated that the RVCL model outperformed existing mainstream DL models, such as AlexNet, VGG, ResNet, ViT, and EfficientNet, in identifying the MTM subtype, showcasing superior diagnostic efficacy. Furthermore, compared to traditional ML models, including LR, RF, and SVM, the RVCL algorithm exhibited significant advantages, while the performance of the three traditional ML models in MTM identification showed no notable differences.

The superior diagnostic performance of the RVCL model over existing DL and ML models can be attributed to its innovative architectural design, which is reflected in three key aspects. First, by leveraging ResNet-50 to extract local features and integrating ViT to capture long-range dependencies, RVCL achieves synergistic modeling of both local and global features. This approach addresses the limitations of traditional CNNs in global context modeling and pure Transformers in local detail capture, significantly improving the Recall metric (external test set Recall of 75%, a notable increase compared to ViT’s 50%). Second, RVCL effectively combines residual learning with self-attention mechanisms. The residual connections mitigate the vanishing gradient problem, while the self-attention mechanism dynamically adjusts feature weights, enabling the model to focus more effectively on critical regions. This results in exceptional performance in F1 Score (78%) and AUC (93%). Finally, RVCL employs a hybrid loss function that combines binary cross-entropy (BCE) loss with triplet loss. The BCE loss ensures classification accuracy, while the triplet loss enhances feature discriminability, allowing the model to maintain high Precision (91%) while significantly improving Recall and F1 Score. This achieves a better balance between precision and recall. These design elements collectively enable RVCL to excel in fine-grained classification tasks.

Traditional CNNs, including architectures like AlexNet, VGG, and ResNet, demonstrate strong specificity, making them effective for excluding non-MTM cases. However, their sensitivity remains suboptimal, and they exhibit notable performance declines in external validation, suggesting susceptibility to overfitting and limited generalizability. ResNet shows relatively stable training performance but struggles with inconsistent precision in new datasets, raising concerns about false positives. ViT, relying on global feature analysis through self-attention, achieves exceptional specificity but suffers from poor sensitivity and imbalanced performance, requiring extensive datasets for optimal training. EfficientNet balances specificity and generalizability better than other baseline models, with a scalable architecture favoring deployment, though its modest sensitivity limits reliability in high-stakes MTM-HCC detection. In contrast, the hybrid RVCL model synergizes local feature extraction and global contextual understanding, delivering robust and balanced performance across sensitivity and specificity, alongside strong generalizability. However, its computational demands may hinder practical implementation in resource-limited environments. The integration of clinical data into RVCL (RVCL + C) further elevates diagnostic precision and sensitivity, achieving near-perfect performance, though reliance on standardized clinical inputs poses challenges in heterogeneous healthcare settings. While traditional CNNs and ViT prioritize specificity at the expense of sensitivity, EfficientNet offers a middle ground with deployability but compromises diagnostic accuracy. RVCL/C+ emerges as the gold standard for comprehensive diagnosis in well-resourced clinical workflows, whereas EfficientNet may suffice for initial screening where computational or data constraints exist. This underscores the importance of aligning model complexity, data quality, and clinical objectives to optimize real-world applicability.

Compared to traditional ML models such as LR, RF, and SVM, the RVCL model demonstrated significantly superior diagnostic performance, while the three traditional ML models showed no notable differences in MTM identification. This finding exhibits some inconsistency with previous studies. For instance, Zhang et al [18], in a single-center study based on 232 magnetic resonance imaging (MRI) images, reported that LR outperformed other traditional ML algorithms (including K-nearest neighbor, Bayesian, decision tree (DT), and SVM), achieving AUC values of 0.766 and 0.739 on the training and test sets, respectively. Additionally, Feng et al [15] extracted radiomics features from contrast-enhanced CT images of 365 patients, selected features using least absolute shrinkage and selection operator (LASSO) regression, and incorporated them into an SVM classifier, yielding AUC values of 0.80 and 0.74 on the internal and external test sets, respectively. Cai et al [23] extracted radiomics features from MRI images of 127 patients and employed an RF algorithm to identify MTM-HCC, achieving AUC values of 0.916 and 0.833 on the training and validation sets, respectively. The discrepancies in these results may stem from differences in patient cohorts, imaging modalities (MRI vs CT), regions of interest (two-dimensional vs three-dimensional), and feature dimensionality reduction methods. In contrast, our study, through a head-to-head comparison, further substantiates the unique advantages of DL models in handling high-dimensional, non-linear medical imaging data, particularly in extracting complex features and modeling global contextual information. Traditional ML models, due to their limited capability in feature extraction, struggle to capture subtle differences in CT images, resulting in relatively modest performance in MTM identification tasks.

Our findings indicate that the elevated serum AFP levels serve as an independent predictor of the MTM subtype, which aligns with previous reports [7, 15]. However, integrating AFP into the RVCL model did not significantly enhance its diagnostic performance (*p* ≥ 0.05), consistent with prior studies [15]. This may suggest that the RVCL model has already extracted sufficient diagnostic information from CT images, rendering the additional clinical information provided by AFP of limited contribution to model performance. However, this does not preclude the potential for further enhancing model performance by incorporating other clinical or imaging features, such as tumor markers, pathological data, or multimodal imaging. The fusion of multimodal data may provide the model with more comprehensive information, thereby enabling more precise diagnosis in complex cases.

Our study has several limitations. Firstly, our sample size—while multi-institutional—remains relatively small for a DL study. Larger multicenter datasets are needed to reduce potential biases and further validate generalizability in order to achieve better performance. Secondly, DL is often seen as a “black box”, and limited model interpretability can reduce clinical adoption. To enhance the interpretability of the model, future research could incorporate interpretability techniques (such as Grad-CAM) to visualize the decision-making process of the model, thereby increasing clinicians’ trust in the model. Lastly, the use of different CT scanners from two centers may affect the radiomics features.

In conclusion, the RVCL model demonstrates significant advantages in the non-invasive diagnosis of MTM-HCC subtypes, leveraging an innovative architecture that synergistically integrates CNNs and ViTs. Its robust diagnostic performance highlights its potential as a clinical decision-support tool, paving the way for future applications in precision medicine and personalized treatment strategies.

## Supplementary information

ELECTRONIC SUPPLEMENTARY MATERIAL

## Data Availability

The datasets used and/or analyzed during the current study are available from the corresponding author upon reasonable request.

## Abbreviations

AUC: Area under the curve
CT: Computed tomography
DL: Deep learning
HCC: Hepatocellular carcinoma
LR: Logistic regression
ML: Machine learning
MRI: Magnetic resonance imaging
MTM: Macrotrabecular-massive
RF: Random forest
SVM: Support vector machine
