# Supplementary material for: Macrotrabecular-massive subtype in hepatocellular carcinoma based on contrast-enhanced CT: deep learning outperforms machine learning
Source: Insights Imaging. 2025 Aug 28;16:186. doi: 10.1186/s13244-025-02063-w (PMC12394088; doi:10.1186/s13244-025-02063-w)
Supplement: Supplementary file 1 — ELECTRONIC SUPPLEMENTARY MATERIAL [file 13244_2025_2063_MOESM1_ESM.pdf]

Macrotrabecular-Massive Subtype in Hepatocellular Carcinoma Based on contrast-enhanced CT: Deep Learning Outperforms Machine Learning

ELECTRONIC SUPPLEMENTARY MATERIAL

Table S1：CT Scanning Parameters for Center 1

Parameters	Somatom Definition Flash(Siemens Healthineers, German)	Somatom Definition AS+(Siemens Healthineers, German)	IQon Spectral CT (Philips Healthcare, Netherlands)
Tube voltage	120 kVp	120 kVp	120 kVp
Tube current	205 mAs	260 mAs	145 mAs
helical pitch	0.6	0.6	1
Rotation time	0.5s	0.5s	0.5s
Detector collimation	128 ×0.6 mm	128×0.6 mm	64 × 0.625 mm
Injection rate	3.0 mL/s	3.0 mL/s	3.0 mL/s
Injection dose	1.5 mL/kg	1.5 mL/kg	1.5 mL/kg
Arterial phase CT	using the automated push-injection tracking technique (15 seconds after the abdominal aortic attenuation value reached 100 HU)	using the automated push-injection tracking technique (15 seconds after the abdominal aortic attenuation value reached 100 HU)	using the automated push-injection tracking technique (15 seconds after the abdominal aortic attenuation value reached 100 HU)
Portal venous phase CT	50–70s after injection	50–70s after injection	50–70s after injection
Delayed phase CT	180s after injection	180s after injection	180s after injection
Image matrix	512 × 512	512 × 512	512 × 512
Reconstruction section thickness	1 mm	1mm	1 mm

Table S2：CT Scanning Parameters for Center 2

Parameters	IQon spectral CT (Philips Healthcare, Netherlands)
Tube voltage	120 kVp
Tube current	250 mAs
helical pitch	0.95
Rotation time	0.50s
Detector collimation	128 ×0.6 mm
Injection rate	3.5 mL/s
Injection dose	1.5 mL/kg
Arterial phase CT	using the automated push-injection tracking technique (25 seconds after the abdominal aortic attenuation value reached 100 HU)
Portal venous phase CT	60–70s after injection
Delayed phase CT	180s after injection
Image matrix	512 × 512
Reconstruction section thickness	1 mm

Appendix S1:Feature Extraction Capture

The task of tumor classification relies heavily on extracting representative features from medical images. CNNs are commonly employed for this task due to their ability to learn hierarchical feature representations. We utilize ResNet-50 as the backbone for feature extraction. The key innovation in ResNet is the introduction of residual connections, which mitigate the vanishing gradient problem that often arises in deep networks. Each residual block in ResNet can be expressed mathematically as follows:

$$y = F(x, \{W_i\}) + x,$$

where $x \in \mathbb{R}^{C \times H \times W}$ represents the input feature map, $F(x, \{W_i\})$ denotes the output after applying convolutional layers and activation functions, and y is the output of the residual block. This design enables the network to approximate identity mappings, thereby preventing overfitting and improving the flow of gradients during training, especially in deeper networks. The ResNet architecture can capture local features from the input image, producing a feature map with dimensions $C \times H \times W$, where C is the number of channels, and H and W are the spatial dimensions of the feature map. To integrate these features with the the next module, we apply a convolution operation to adjust the channel size and create feature embeddings that are compatible with the second component

$$x_{ResNet} \in \mathbb{R}^{D \times H \times W}$$

where D is the embedding dimension, typically set to align with the ViT's input.

Blocker Relation Capture

While ResNet excels at extracting local features, global contextual information is equally important for tumor classification. To address this, we incorporate the ViT, which captures long-range dependencies through self-attention mechanisms. In ViT, the input image is divided into non-overlapping patches, each of size $p \times p$, and each patch is flattened into a one-dimensional vector. These patch embeddings are then fed into the transformer layers for contextual modeling. Let x_{ResNet} be the feature map produced by ResNet. The image is divided into n patches, each of size $p \times p$, and the flattened patch embeddings are computed as:

$$x_{patch}^{i} = Flatten(x_{ResNet}[i])W_{patch} + b_{patch}, for\ i = 1, \cdots, n,$$

where $W_{patch} \in \mathbb{R}^{p^2C\times D}$ is the learnable projection matrix, and b_{patch} is the bias term. Each $x_{patch}^{i} \in \mathbb{R}^{D}$ is a vector representing a patch from the feature map, and these vectors are fed into the self-attention mechanism to model the global dependencies. The core of ViT is the self-attention mechanism, which computes the relationship between each pair of patches. The attention operation can be mathematically formulated as:

$$Attention(Q, K, V) = softmax\left(\frac{QK^T}{\sqrt{d_k}}\right)V$$

where Q, K, and V represent the query, key, and value matrices, respectively, derived from the patch embeddings. The softmax operation ensures that the attention weights are normalized, allowing the model to focus on relevant parts of the image. After stacking multiple layers of self-attention and feed-forward networks, ViT produces a sequence of feature embeddings of size $n \times D$, where n is the number of patches. To obtain a fixed-size representation of the image, we apply global average pooling over the patch embeddings:

$$z_{global} = \frac{1}{n}\sum_{i=1}^{n} z_{ViT}[i]$$

This results in a global feature vector $z_{global} \in \mathbb{R}^{D}$, which encapsulates the global context of the image, ready to be passed to

the classification head.

Classification Head and Loss Function

Once the global feature representation is obtained, we pass it through a fully connected layer to perform the final classification. Let $z_{global} \in \mathbb{R}^D$ be the input to the classification head, which consists of two linear transformations:

$$h_1 = ReLU(W_1 z_{global} + b_1)$$

$$h_2 = W_2 h_1 + b_2$$

Here, $W_1 \in \mathbb{R}^{D\times H}$ and $W_2 \in \mathbb{R}^{H\times 1}$ are trainable weight matrices, and b_1, b_2 are the corresponding bias terms. The output $h_2 \in \mathbb{R}^1$ is passed through a sigmoid activation function to predict the probability of the tumor being malignant:

$$p_{malignant} = \sigma(h_2),$$

where $\sigma(x) = \frac{1}{1+e^{-x}}$ is the sigmoid function, and $p_{malignant} \in [0, 1]$ represents the probability of malignancy. To train the model, we use a hybrid loss function that combines binary cross-entropy (BCE) loss for classification and triplet loss for enhancing feature separability. The binary cross-entropy loss is given by:

$$\mathcal{L}_{BCE} = -\frac{1}{N}\sum_{i=1}^{N}[y_i \log(p_{malignant}) + (1 - y_i)\log(1 - p_{malignant})]$$

where y_i is the ground truth label, $p_{malignant}$ is the predicted probability, and N is the batch size. This loss function encourages

the model to predict the malignancy status correctly.

Additionally, we introduce triplet loss to promote better feature discrimination. Given an anchor sample x_a, a positive sample x_p(random sample which has the same class with anchor sample), and a negative sample x_n(random sample which has the different class with anchor sample), the triplet loss is defined as:

$$\mathcal{L}_{triplet} = \max\left(\left\|f(x_a) - f(x_p)\right\|_2^2 - \|f(x_a) - f(x_n)\|_2^2 + \alpha, 0\right)$$

Where $f(x)$ represents the feature embedding of the sample, $\|\cdot\|_2$ is the Euclidean distance, and α is a margin hyperparameter. The total loss function is a weighted sum of the binary cross-entropy and triplet loss,

$$\mathcal{L}_{total} = \mathcal{L}_{BCE} + \lambda \cdot \mathcal{L}_{triplet}$$

where λ is the weight coefficient that balances the two loss terms.

By incorporating this learning framework, we are able to achieve accurate tumor classification while simultaneously learning more discriminative feature representations, improving both classification accuracy and model generalization.

Appendix S2: Machine Learning Feature Selection Results

For each region of interest (ROI), 1,407 features were extracted. Since two ROIs (arterial phase and portal venous phase) were analyzed per patient, a total of 2,814 features were obtained per patient. These included: 210 original features，1488 Wavelet

Transform features，372 Square features，186 Logarithm features，186 Laplacian of Gaussian features，186 Gradient features，186 Exponential features.During feature selection, 1,688 radiomics features with both intra- and inter-observer intraclass correlation coefficients (ICC) > 0.80 were retained. We performed dimensionality reduction using the Pearson correlation coefficient and feature selection via analysis of variance (ANOVA). The final selected features for classification were:

Logarithm_glcm_ClusterShade_A,

Exponential_firstorder_Median_A,

Logarithm_glszm_GrayLevelVariance_A,

Logarithm_firstorder_Variance_A,

Exponential_firstorder_RobustMeanAbsoluteDeviation_V,

Wavelet-LLH_glcm_Imc2_V,

Logarithm_firstorder_90Percentile_V, ,

Exponential_glcm_ClusterShade_V,

hese features were used for classification with three machine learning classifiers(random forest, support vector machines, and logistic regression).

Table S3

Radiomic Features Used in This Study

Category	Feature
Shape(n=12)	Elongation,MajorAxisLength, Maximum2DDiameterColumn, Maximum2DDiameterRow, Maximum2DDiameterSlice, Maximum3DDiameter, MeshVolume, MinorAxisLength, Sphericity, SurfaceArea, SurfaceVolumeRatio, VoxelVolume
First-order(n=270)	10Percentile, 90Percentile, Energy, Entropy, InterquartileRange, Kurtosis, Maximum, MeanAbsoluteDeviation, Mean, Median, Minimum, Range, RobustMeanAbsoluteDeviation, RootMeanSquared, Skewness, TotalEnergy, Uniformity, Variance
GLCM(n=360)	Autocorrelation, ClusterProminence, ClusterShade, ClusterTendency, Contrast, Correlation, DifferenceAverage, DifferenceEntropy, DifferenceVariance, Id, Idm, Idmn, Idn, Imc, InverseVariance, JointAverage, JointEnergy, JointEntropy, MCC
GLRLM(n=240)	GrayLevelNonUniformity, GrayLevelNonUniformityNormalized, GrayLevelVariance, HighGrayLevelRunEmphasis, LongRunEmphasis, LongRunHighGrayLevelEmphasis, LongRunLowGrayLevelEmphasis, LowGrayLevelRunEmphasis, RunEntropy, RunLengthNonUniformity, RunPercentage, RunVariance, ShortRunEmphasis, ShortRunHighGrayLevelEmphasis, ShortRunLowGrayLevelEmphasis
GLDM(n=210)	DependenceEntropy, DependenceNonUniformity, DependenceNonUniformityNormalized, DependenceVariance, GrayLevelNonUniformity,

	GrayLevelVariance, HighGrayLevelEmphasis, LargeDependenceEmphasis, LargeDependenceHighGrayLevelEmphasis, LargeDependenceLowGrayLevelEmphasis, LowGrayLevelEmphasis, SmallDependenceEmphasis, SmallDependenceHighGrayLevelEmphasis, SmallDependenceLowGrayLevelEmphasis
GLSZM(n=240)	GrayLevelNonUniformity, GrayLevelNonUniformityNormalized, GrayLevelVariance, HighGrayLevelZoneEmphasis, LargeAreaEmphasis, LargeAreaHighGrayLevelEmphasis, LargeAreaLowGrayLevelEmphasis, LowGrayLevelZoneEmphasis, SizeZoneNonUniformity, SizeZoneNonUniformityNormalized, SmallAreaEmphasis, SmallAreaHighGrayLevelEmphasis, SmallAreaLowGrayLevelEmphasis, ZoneEntropy, ZonePercentage, ZoneVariance, GrayLevelNonUniformity, GrayLevelVariance, HighGrayLevelZoneEmphasis, LargeAreaEmphasis, LargeAreaHighGrayLevelEmphasis, LargeAreaLowGrayLevelEmphasis,
NGTDM(n=75)	Busyness, Coarseness, Complexity, Contrast, Strength

Note. GLCM= Gray-Level Co-occurrence Matrix, GLRLM= Gray-Level Run-Length Matrix, GLSZM= Gray-Level Size Zone Matrix, GLDM=Gray-Level Dependence Matrix, NGTDM= Neighboring Gray-Tone Difference Matrix

Table S4：Univariate Logistic Regression Analysis for Prediction of Macrotrabecular massive Hepatocellular Carcinoma in the Training Dataset

Characteristics	Univariate Analysis	
	OR(95%CI)	*P* Value
Age > 50 y	0.816(0.381-1.746)	0.600
Sex (Male)	0.792(0.402-1.558)	0.499
AFP level >100 ng/mL	1.001(1.000-1.002)	0.027
Platelet count > 100*109/L	0.975(0.468-2.029)	0.946
Neutrophil count (^9/L)	1.001(0.912-1.099)	0.984
Lymphocyte count (^9/L)	0.724(0.405-1.294)	0.276
The neutrophil-to-lymphocyte ratio	0.997(0.946-1.05)	0.896
Prothrombin time > 13 s	1.263(0.653-2.446)	0.488
INR > 1.15	1.247(0.647-2.403)	0.510
AST level > 40 U/L	1.218(0.623-2.381)	0.565
ALT level > 50 U/L	1.020(0.526-1.978)	0.952
Albumin level > 40 g/L	0.877(0.407-1.89)	0.739
Total bilirubin level > 19 umol/L	1.330(0.664-2.667)	0.421
Size > 5 cm	1.219(0.623-2.384)	0.563

Note- AFP = α-fetoprotein, INR=International normalized ratio, ALT=alanine aminotransferase, AST=aspartate aminotransferase

Table S5: Performance of the Machine learning Models for Predicting Macrotrabecular massive Hepatocellular Carcinoma

Model	Training Set(n=198)						Internal Test Set(n=85)						External Test Set(n=85)					
	Acc	Sen	Spe	Precision	F1 Score	AUC	Acc	Sen	Spe	Precision	F1 Score	AUC	Acc	Sen	Spe	Precision	F1 Score	AUC
LR	0.56	0.57	0.56	0.29	0.38	0.53[0.44,0.62]	0.71	0.27	0.86	0.40	0.32	0.49[0.34,0.64]	0.53	0.85	0.39	0.37	0.52	0.60[0.48,0.74]
RF	0.39	0.87	0.25	0.26	0.41	0.53[0.43,0.61]	0.66	0.36	0.76	0.35	0.35	0.50[0.36,0.64]	0.54	0.58	0.63	0.39	0.47	0.49[0.39,0.63]
SVM	0.63	0.45	0.69	0.31	0.37	0.54[0.45,0.64]	0.36	0.91	0.17	0.28	0.43	0.45[0.31,0.58]	0.55	0.77	0.46	0.37	0.50	0.59[0.46,0.72]

Note.-Data in parentheses are 95% CIs. Acc= accuracy,AUC=area under the receiver operating characteristics curve, Sen= sensitivity, Spe= specificity.
